# Observations and recommendations on the investigation of clinical decision-making and usage involving electrophysical agents

**DOI:** 10.1186/s13584-015-0050-7

**Published:** 2015-10-08

**Authors:** David M. Selkowitz

**Affiliations:** Department of Physical Therapy, MGH Institute of Health Professions, 36 1st Avenue, Charlestown Navy Yard, Boston, MA 02129 USA

**Keywords:** Electrophysical agents, Electrotherapy, Clinical decision-making, Physiotherapy

## Abstract

Electrophysical agents (EPAs), including electrotherapy, are important components of patient/client management. A recent study by Springer et al. has elucidated the pattern of use and factors in clinical decision-making by Israeli physiotherapists regarding EPAs. It is evident from their data that EPAs, especially those related to electrotherapy, are still considered relevant to physiotherapy practice. Included in this commentary are observations on the findings of the current study, as well as recommendations on an alternative approach to the investigation of clinical decision-making and usage of EPAs. Discussion also includes a proposed and apparent de-emphasis of EPAs in physiotherapy education and practice in the USA, which may impact some of the factors found in the current study to be relevant in clinical decision-making and usage for EPAs.

## Commentary

### Background and observations on the current study

The current study by Springer et al. [1] has investigated the contribution of various factors to clinical decision-making regarding the application of electrophysical agents (EPAs). My area of experience within EPAs is primarily in electrotherapy, and my commentary will be focused in this area. According to the results of the current study, the greatest availability and frequency of use of EPAs, apart from heat packs and ultrasound, was primarily in the electrotherapeutic EPAs.

This study demonstrates that EPAs are considered relevant to physiotherapy practice and used regularly by physiotherapists in Israel, although only heat packs, interferential current (IFC), and transcutaneous electrical nerve stimulation (TENS) are used daily by as many as approximately 50 % of the study’s participants. The lesser usage of some EPAs may be related to availability, although cold packs and ice had high availability but relatively low usage. Of the electrotherapeutic EPAs, the least used was functional electrical stimulation (FES). These may be due to the fact that the greatest number of physiotherapists participating in the study were practicing in the area of orthopedic and sports rehabilitation (62.5 %), in which there is typically not much indication for FES; as opposed to neurologic rehabilitation (20.1 %), the area in which FES is more often used.

The factors with the greatest frequencies of “strong” or “very strong” influence on the use of EPAs appeared to be previous clinical experience, availability of equipment, and degree of self-confidence operating the device, with frequencies between approximately 60–80 %. It would seem that greater availability would lead to more clinical experience, which in turn would lead to greater self-confidence. The factors with next highest levels of influence, with frequencies between 50 and 60 %, were entry-level and continuing education, as well as research evidence for efficacy. I would consider it to be a more desirable outcome for these factors to be much higher. I am unfamiliar with education and practice in Israel where this study and its participants are based. However, there are potential factors and issues in the United States that would explain results that would be disappointing in these areas, which will be discussed later in the commentary. This study also found that the preferences of the patient do not seem to play a major role in clinical decision-making regarding EPAs. This seems disappointing given the importance placed on it within the framework of evidence-based practice. However, it may be that clinicians do not believe that most patients are well-informed regarding EPAs compared to the clinicians themselves, enough to make an appropriate decision on incorporating EPAs into their own treatment program. This also may not be very different from the influence of patient preference on other intervention choices. Perhaps this can be addressed through patient education on EPAs, by physiotherapists.

The current study has investigated numerous EPAs. This is a large endeavor that covers a lot of ground, but it may sacrifice some of the details that can help clarify specific issues related to patterns of use of smaller or individual categories (as they were defined in the study) of EPAs. This might prevent results that could be misleading in the low or high extremes.

### Issues in the investigation of clinical decision-making and usage

The current study has investigated electrotherapy with respect to device classifications, i.e., FES, neuromuscular electrical stimulation (NMES), TENS, and IFC, rather than interventions or treatment purposes. This is appropriate if one is interested in the use of types of devices. The first three have come to be synonymous with particular treatment purposes. FES is associated with the use of electrotherapy for orthotic substitution for function, such as stimulating dorsiflexion during the swing phase of gait. NMES has come to be associated with treatment purposes for enhancing muscle performance such as strengthening, while TENS is a term that has long been considered synonymous with pain control. However, these classifications or terms can be sub-divided into other purposes, as well. In addition, multiple types of currents and waveforms may be used within some of these classifications. For example, NMES may include biphasic pulsatile current as well as medium frequency burst-modulated alternating current. Even IFC has been found to be effective for NMES purposes such as muscular force production [2].

Therefore, I would appreciate seeing the investigation of electrotherapy, (actually, all EPAs) based on the treatment purpose, at least in addition to if not more so than a device classification. The authors stated that they included questions in their questionnaire, not reported in the current paper, that were related to indications for the use of specific electrical current forms, which may address my concern to some degree. First, the problem to be treated must be determined, then one can proceed with the treatment purpose application (e.g., strengthening). With electrotherapy, the treatment purpose applications must be based on identifying the neurophysiologic response that is necessary (e.g., muscle contraction, for strengthening), and then selecting the appropriate parameter settings that will create the desired response at the appropriate level (e.g., a frequency of 50 pps to create a tetanic contraction, with high enough current amplitude and current duration, or charge, for a strong contraction) [3, 4].

Devices of a particular type or classification may have multiple and varied treatment purpose applications [3]. For example, a TENS device can be used for purposes other than pain control, such as enhancement of circulation. Similarly, the device classifications used in the current study have overlapping treatment purpose applications. For example, both IFC and TENS devices may be used for pain control. Therefore, frequency of use (as investigated in the current study) of one type of device might subtract from that of another device. Conversely, it could add to the frequency of use based on treatment purpose.

If clinicians are using one type of device over another for a treatment purpose for which either type of device could be used, it would be useful to know this as well as the reasons for it. On the other hand, it would be useful to know if the clinician fully understands that a type of device has multiple treatment purpose applications. If not, the choice of device might be related to the clinician not fully understanding the available appropriate options. The authors of the current study point out that availability might have meant that the device might not be available when needed. However, if clinicians were aware that a particular device has multiple treatment uses, they might opt to use a different device that could fulfill the same purpose as the originally desired device if the latter device was currently being used by another clinician in their facility. It would also be useful to know the frequency of use of electrotherapy (or any physical agent, for that matter) by treatment purpose when the clinician has identified the treatment purpose and that there was another option for treatment, and whether or not more than one of the treatment options were chosen, as well as the reasons for the clinician’s selections.

### Apparent de-emphasis of EPAs in education and practice

The current study has shed some light on the pattern of use of electrotherapy and other physical agents. This comes at a point in the evolution of the field of physiotherapy in which some are suggesting or actually putting into practice the de-emphasis of EPAs, including electrotherapy, by physiotherapists. Recently the APTA has made a strong recommendation to this effect [5]. In addition, there appears to be a de-emphasis of this area of the field in entry-level physiotherapy education curricula in the United States. Some physiotherapy programs have limited the number of hours for courses on EPAs, with some programs allotting only limited hours for this content within courses on other content. These sentiments and practices may be associated with long-held biases regarding overuse of physical agents without proper application of clinical decision-making by physiotherapists and use of other, additional treatment interventions. This might have been an issue more than 25 years ago, but is not based on sound evidence now [6, 7]. There may also be a misconception that the evidence for use of electrotherapy is minimal or non-existent. In addition, electrotherapy and other physical agents have been pejoratively referred to as passive interventions. However, other well regarded interventions such as joint manipulation are passive, and the “passiveness” of the intervention is not a problem if the intervention is appropriate and applied appropriately. In any case, electrotherapy and other physical agents, as with most if not all interventions, are rarely recommended to be used exclusively, unless other interventions would be inappropriate or less appropriate and provide no additional advantage.

Recommendations for inappropriate limited use or non-use have the further effect of limiting physiotherapist student and clinician understanding and ability to appropriately apply electrotherapy and physical agents. These further feed into rationales for limited and non-use. This cycle may lead to a predisposition resulting in the removal of EPAs from the physiotherapist’s armamentarium of interventions.

## Conclusions

The current study speaks to the use of EPAs in Israel by physiotherapists and provides data that can be built upon to improve continued appropriate use. EPAs appear to be considered relevant to physiotherapist practice in Israel. I have provided recommendations for future study of the use of electrotherapy and EPAs, including investigations based on treatment purpose. The authors of the current study reported that they have additional data, that they can report on in the future, which may address these recommendations.

## References

[1] Springer S, Laufer Y, Elboim-Gabyzon M (2015). Clinical decision making for using electro physical agents by physiotherapists, an Israeli survey. Isr J Health Policy Res.

[2] Bellew JW, Beiswanger Z, Freeman E, Gaerte C, Trafton J (2012). Interferential and burst-modulated biphasic pulsed currents yield greater muscular force than Russian current. Physiother Theory Pract.

[3] Selkowitz DM, Cameron MH (1999). Electrical currents. Physical agents in rehabilitation: from research to practice.

[4] Selkowitz DM, Rossman E, Fitzpatrick S (2009). Effect of burst-modulated alternating current carrier frequency on current amplitude required to produce maximally-tolerated electrically-stimulated quadriceps femoris knee extension torque. Am J Phys Med Rehabil.

[5] White NT, Delitto A, Manal TJ, Miller S (2015). The American Physical Therapy Association’s top five choosing wisely recommendations. Phys Ther.

[6] Belanger AY, Cameron MH, Michlovitz SL, Bellew JW, Freeman L (2015). On “The American Physical Therapy Association’s top five choosing wisely recommendations”. White NT, Delitto A, Manal TJ, Miller S. Phys Ther. doi: 10.2522/ptj.20140287. Phys Ther.

[7] Bjordal JM, Ronzio O, Baxter GD, Sluka KA (2015). On “The American Physical Therapy Association’s top five choosing wisely recommendations”. White NT, Delitto A, Manal TJ, Miller S. Phys Ther. doi: 10.2522/ptj.20140287. Phys Ther.

